# PRISM (Pictorial Representation of Illness and Self-Measure) as Visual Tool to Support Oral Health Education Prior to Endoprosthetic Joint Replacement—A Novel Approach in Dentistry

**DOI:** 10.3390/jcm11092508

**Published:** 2022-04-29

**Authors:** Gerhard Schmalz, Laura Schmidt, Rainer Haak, Stefan Büchi, Szymon Goralski, Andreas Roth, Dirk Ziebolz

**Affiliations:** 1 Department of Cariology, Endodontology and Periodontology, University of Leipzig, 04103 Leipzig, Germany; laura.schmidt@medizin.uni-leipzig.de (L.S.); rainer.haak@medizin.uni-leipzig.de (R.H.); dirk.ziebolz@medizin.uni-leipzig.de (D.Z.); 2 Clinic for Psychotherapy and Psychosomatics “Hohenegg”, 8706 Meilen, Switzerland; stefan.buechi@hohenegg.ch; 3 Department of Orthopaedics, Trauma and Plastic Surgery, University Hospital Leipzig, 04103 Leipzig, Germany; szymon.goralski@medizin.uni-leipzig.de (S.G.); andreas.roth@medizin.uni-leipzig.de (A.R.)

**Keywords:** endoprosthesis, joint replacement, visual metaphor, patient education

## Abstract

Objective: This study aims to evaluate the application of Pictorial Representation of Illness and Self-Measure (PRISM) in educating patients regarding oral health before endoprosthesis (EP). Methods: The study consisted of two parts: (I) a cross-sectional study, where patients received a PRISM interview, oral health briefing and oral examinations (treatment need, oral focus). (II) In an observational part, patients were randomly assigned to either PRISM task (Test) or flyer-based verbal briefing (Control). Before and after the interviews, patients answered a questionnaire regarding importance of oral health for EP. Results: (I) 122 patients were included. The distance between subject (“myself”) and objects (oral health issues or EP) in the PRISM task were mainly not associated with age, gender, and oral conditions. In part (II), 80 patients (PRISM: *n* = 40, Control: *n* = 40) were included. After the interview, the values for perceived relationship between EP and teeth (*p* < 0.01), EP and gums (*p* < 0.01), and EP and dental consultations (*p* < 0.01) significantly increased in both groups. Both groups perceived a high benefit of the interview and felt well educated. Conclusions: PRISM has comparable positive effects like a flyer-based verbal briefing. PRISM as a novel visual tool can support the patient education regarding oral health before EP.

## 1. Introduction

Information and education of patients with regard to oral health and potentially related systemic diseases and conditions play an integrative role in improving healthcare [1]. Oral diseases, which are highly prevalent all over the world, are estimated to be associated with hundreds of systemic diseases and medications [2]. Thereby, it is conspicuous that awareness of patients about this relationship and potential consequences for prevention and therapy of their oral and systemic diseases is often low; e.g., diabetic patients have limited knowledge and attitudes regarding their oral health, although the bidirectional relationship between periodontitis and diabetes is already known for decades [3,4]. This phenomenon is not limited to patients with chronic non-communicable diseases. Even patients with a generally increased health behavior like pregnant women were found to have an inappropriate knowledge and awareness regarding oral health [5]. A systematic review highlighted the health-practitioner communication to be crucial for a sufficient patient education, whereby dentists and health professionals are the main source of information for the patients [1]. While evidence for successful patient education and motivation in context of oral-systemic interaction is limited, another review article implicated that scientific research in this field would be strongly needed [6].

Pictorial Representation of Illness and Self-Measure (PRISM) is a visual tool, based on a metaphor, which induces a self-reflection process and helps patients and practitioners as a basis for communication and therapy [7]. This method was initially developed to measure illness impact and suffering of patients [8,9]. However, meanwhile it has a broad field of application, e.g., in context of alcohol addiction [10], risks related to travelling [11] or to assess work-related stress in anesthesiologists [12]. Moreover, PRISM has been shown to be beneficial in education of patients with rheumatoid arthritis, by improving patient–clinician communication and self-efficiency; furthermore, it induced resource activation [13]. All mentioned aspects make PRISM a potentially interesting tool for patient education in dentistry.

One group of certain interest in this context are patients prior to implantation of an endoprosthesis (EP) for joint replacement. Although the evidence is still limited, oral diseases, especially those with infectious aspects are supposed to cause infectious complications after EP [14,15,16]. It has been shown that patients prior to EP implantation have a high need of dental care, making comprehensive oral examination and therapy potentially recommendable in those individuals [17]. Based on the high number of EP placed each year, and the enormous morbidity of patients with EP infections, the risk reduction for infectious complications is crucial in those individuals [18]. Therefore, informing and educating patients prior to EP for healthy oral conditions, sufficient oral hygiene and regular dentist consultations appear a reasonable and valuable approach to ameliorate outcome of EP.

Accordingly, this current study applied PRISM in patients prior to EP implantation for the first time in dentistry. Two parts of the current study had two distinct aims: (I) a cross-sectional study should reveal whether subjectively perceived impact of oral health and EP, as assed with PRISM, would be related to clinical oral conditions of the patients. (II) an observational study should investigate if PRISM leads to increased awareness for the importance of oral health for the health of the future EP. Thereby, PRISM should be examined in comparison to an information-flyer-based education briefing as control measure. It was hypothesized that (I) PRISM findings would be associated with clinical oral health conditions and (II) both PRISM and verbal briefing would lead to a remarkable increase of awareness for oral health in patients before EP implantation.

## 2. Methods

This current study consisted of two parts, (I) a cross-sectional study of patients before EP implantation and (II) an observational study. All study parts were performed in full accordance with the Helsinki Declaration and the study protocol has been reviewed and approved by the local ethics committee (116/20-ek). All participating patients were informed both, verbally and in writing about the study and gave their written informed consent.

### 2.1. Patients

Patients, which were planned for an EP implantation in the clinic for orthopedic surgery were informed about the opportunity to undergo a dental check-up and educational interviews within a clinical study. For this, participating patients were allocated to the Dental University Clinic for dental examination. The following inclusion criteria were formulated prior to the study: age of at least 18 years, necessity of EP implantation of knee or hip joint, the participation war voluntary. Patient-specific exclusion criteria were worse general health, which did not allow examination, insufficient German language, which would not allow the understanding of the interview contents and cognitive impairments, e.g., dementia. Prior to examination, patients were informed about the course and content of the study and gave their written informed consent for participation.

For part (I), the current study aimed to include as many patients as possible to get an overview on PRISM findings related to oral health issues in this specific cohort. For part (II), a sample size estimation was performed. Because the expected effect size could not be predicted due to the absence of comparable literature in the field, no exact sample size calculation was possible. Therefore, the estimation was based on most comparable research in the field [13] and on literature, regarding oral health education of patients with special needs [1]. While a previous RCT with a similar study question included 26 patients in the PRISM group [13], sample size for oral health education studies was much higher [1]. Accordingly, a sample size higher than 26 patients, approximately 35–40 patients each group was striven for the current study.

### 2.2. Oral Examination

All participants received a dental and periodontal examination by experienced and calibrated dentists (kappa > 0.8). The oral examinations were performed under standardized conditions. The number of remaining teeth was assessed visually. Carious teeth, i.e., teeth showing a cavitation of the tooth surface were assessed using mirror and probe. If patients had one or more carious teeth, dental treatment need was recorded. To evaluate periodontal treatment need, the periodontal screening index (PSI) [19,20] was assessed using a periodontal probe (PCP 15/11.5B6, Hu-Friedy, Chicago, IL, USA). Thereby, the presence of PSI score 3 (periodontal probing depth ≥ 3.5 < 5.5 mm) in at least two sextants or PSI code 4 (periodontal probing depth ≥ 5.5 mm) in at least one sextant was recorded as periodontal treatment need. The clinical examination, combined with available radiographs of the patients was used to evaluate if patients had a dental focus with a potential risk of EP infection of oral origin. Those potential oral foci were, e.g., severe periodontitis (suppuration, generalized periodontal probing depth of 6 mm or higher, combined perio-endo lesion), profound caries touching the pulp chamber, severely destroyed teeth or remaining roots, any radiographic signs of periapical inflammation, pericoronally inflamed, retained wisdom teeth, or inflammation of the jaw bone or sinus maxillaris.

### 2.3. PRISM and Verbal Briefing

**Figure 1:**
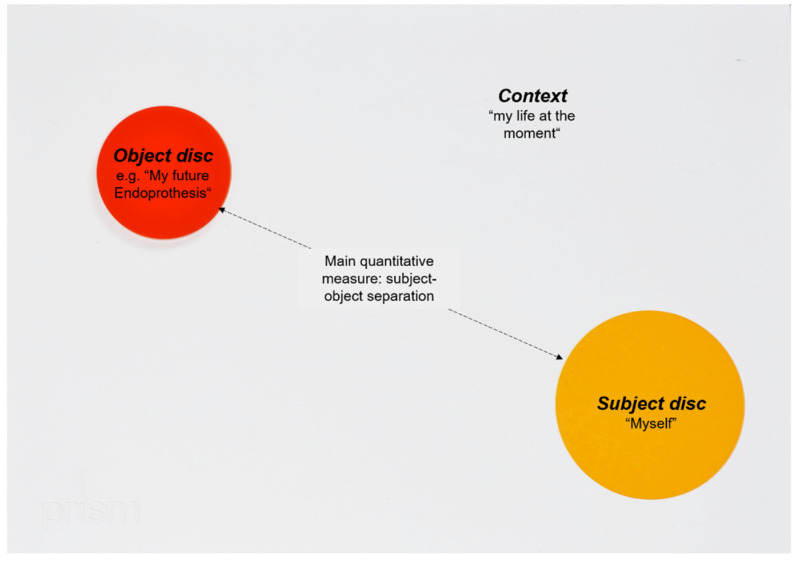
Principle of PRISM. The placement of the objects visualizes the relationship between subject and objects and is able to initiate a reflection process mod. [7].

**Figure 2:**
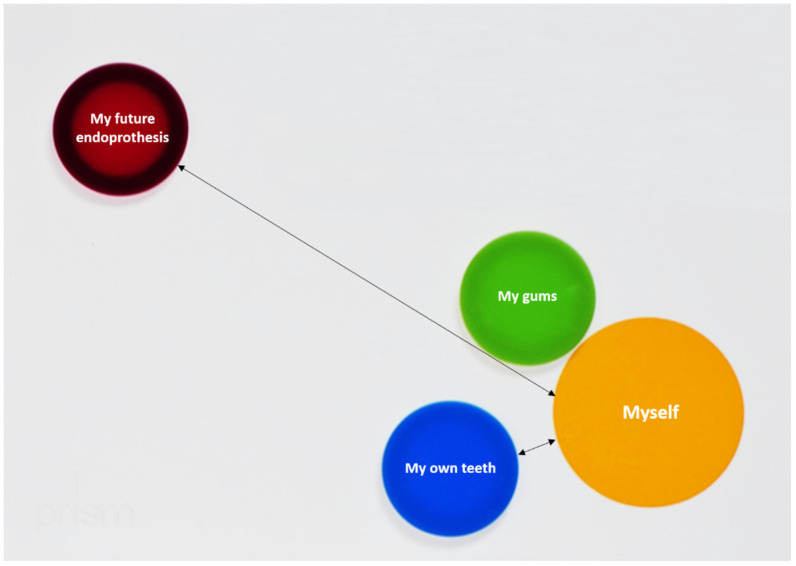
This figure shows the placement of different objects by a patient. The distance between the different objects and the subject was measured in millimeter. When own teeth were placed closer than the EP, like in this case, the respective cluster was rated.

PRISM is a visual metaphor, which has been introduced to quantify suffering of patients [9]. Within this visual metaphor, the relationship between a fixed subject and different objects is displayed in a previously defined context. The context is represented by a white metal board (210 × 297 mm), whereby this context was “my life at the moment” in the current study. In the bottom right-hand corner of the board, a fixed yellow circle (7 cm in diameter) represents the Subject (“myself”). Differently colored magnetic disks with a diameter of 5 cm represent the Objects, reflecting oral health- and EP-related issues (see Figure 1 and Figure 2). In the current PRISM task, patients were instructed to place each Object disk on the board to reflect their perceived importance of their teeth, gums, and future EP. Moreover, the role of dental consultations was evaluated in this context. Within this task, patients reflected their perceived relationship between future EP and their oral health. As a quantitative output generated by PRISM, the distance between Subject and Object was measured. For part (I) of the current study, all patients received such a PRISM interview prior to their oral examination. For part (II), participants received either a PRISM task (Test) or a verbal briefing (Control). The verbal briefing was performed based on an information flyer of the clinic, which summarized the importance of oral conditions for the avoidance of infectious complications of EP. This briefing consisted of important information on the potential risk of oral disease-induced infectious complications of the EP. Thereby, the importance of healthy oral conditions, appropriate oral hygiene, and regular dental visits was explained. Both interventions, the PRISM task as well as flyer-based verbal briefing, lasted for approximately 10 min each (range 8–13 min).

### 2.4. Questionnaires

For the assessment of potential effects of PRISM and flyer-based verbal briefing in part (II), a questionnaire was composed. This questionnaire consisted of 11 questions before and 14 questions after the briefing or PRISM task, respectively. The questions were related to the patient-perceived relationship between their selves and their oral health as well as EP and their perceived importance of oral health for the future EP. All questions were answered on a numerical scale between 0 and 5, whereby higher values reflect higher agreement with the formed statements. The questions were composed by experienced PRISM interviewers and then discussed with dentists and patients to ensure clarity and a good understandability for the patients.

### 2.5. Study Flow

**Figure 3:**
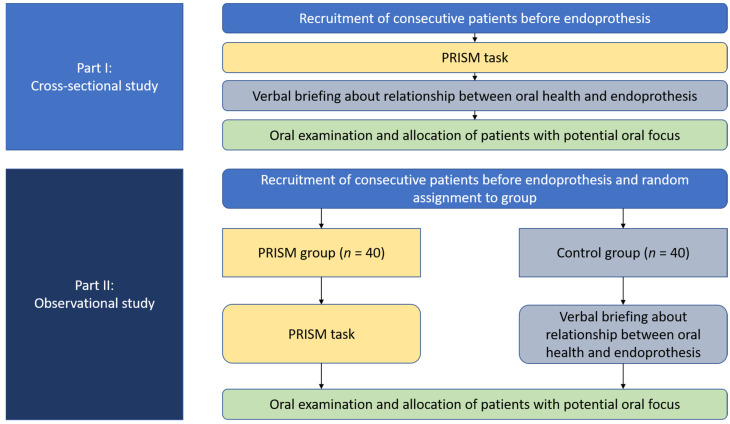
Study flow of both parts of the current investigation.

An overview on the whole study flow is displayed in Figure 3. For part (I), consecutive patients before EP implantation were recruited between September 2020 and April 2021. Those patients received a PRISM task, as described above. Furthermore, these patients underwent the whole oral examination and were informed about the importance of oral health for their future EP. Patients, who had a potential oral focus, were allocated to their dentist for therapy prior to EP implantation.

For part (II), patients before EP implantation were recruited between May 2021 and October 2021. By the drawing of lots, patients were randomly assigned into either PRISM (Test group) or flyer-based verbal briefing (Control group). At first, patients received an 11-questions-containing questionnaire prior to PRISM or verbal briefing, respectively. Subsequently, patients solved either the PRISM task or received the verbal briefing based on the information flyer for oral health of patients before EP implantation. Patients in PRISM group did only solve the PRISM task, without any specific information or briefing about the importance of oral health in context of EP. All interviews were performed by the same experienced interviewer, which had received a comprehensive methodological training with the PRISM task before. After either PRISM or verbal briefing, all participants received the second, 14-questions-containing questionnaire. Afterwards, all patients received an oral examination and were handled as described for part (I).

### 2.6. Statistical Analysis

The statistical analysis was performed using SPSS for Windows, version 24.0 (SPSS Inc., Chicago, IL, US). Kolmogorov–Smirnov test was applied to test the sample for normal distribution, showing a non-normal distribution of all the tested variables (*p* < 0.05). PRISM data were quantitively analyzed for part (I) by considering the absolute distance between objects and subject in millimeter. Moreover, three cluster were determined, i.e., “same importance of EP and teeth”, if teeth and EP had the same distance to the subject, “teeth more important than EP”, if the teeth were placed closer to the subject than the EP and “EP more important than teeth”, if the EP was placed closer to the subject than the teeth. Two independent samples were compared using Mann-Whitney-U test. For comparing more than two independent variables, Kruskal-Wallis test was applied. Categorical data were compared by chi-square or Fisher test, respectively. Two dependent variables were compared by Wilcoxon test.

## 3. Results

### 3.1. Part I—Cross-Sectional Study (n = 122)

#### 3.1.1. Patients

A total of 122 patients, with a mean age of 66.4 ± 11.4 years were included. One quarter (24.6%) had dental treatment need (i.e., presence of at least one carious teeth), the majority of participants showed periodontal treatment need (i.e., PSI score 3 in at least two sextants or PSI code 4 in at least one sextant) (86.1%). A total of 43.4% had a potential dental focus (Table 1).

#### 3.1.2. PRISM Findings

**Figure 4:**
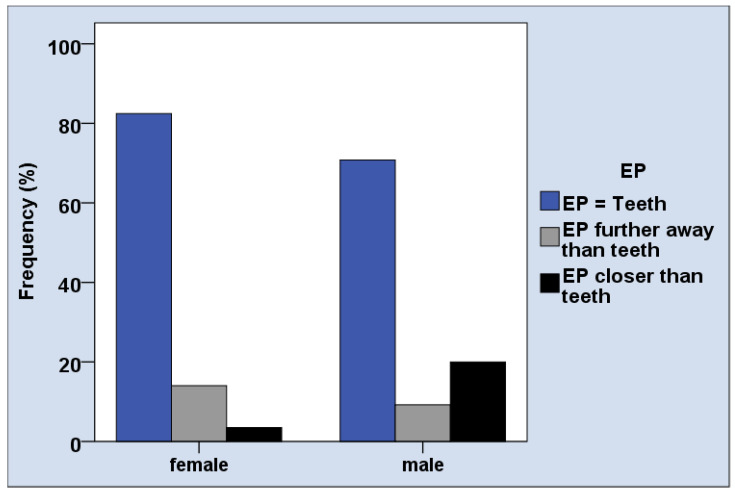
Gender difference in perceiving the relationship between EP and teeth in the PRISM task.

The distances between objects and subject on the PRISM board were small, with an average distance between 11.0 and 13.5 mm, depending on the question. The majority of patients, i.e., 76.2% perceived the same importance of EP and their teeth (Table 2). The distance between subject (“Myself”) and objects (oral health issues and EP) in the PRISM task was mainly not associated with age, gender, and oral conditions (Table 3). Male participants perceived the EP more often closer to the subject than female patients (Table 3, Figure 4, *p* = 0.02). The importance of healthy teeth rated in PRISM was associated with the number of teeth (*p* = 0.04, Table 3), which was not confirmed in post-hoc analysis.

### 3.2. Part II—Interventional Study (n = 80)

#### 3.2.1. Patients

Both groups, PRISM and Control (flyer-based verbal briefing) consisted of 40 patients. Between groups, age, gender, and oral conditions were comparable (*p* > 0.05, Table 4).

#### 3.2.2. Questionnaires

**Figure 5:**
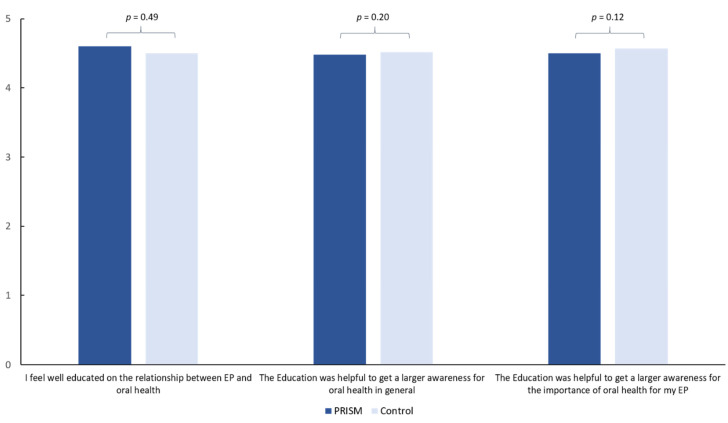
Perceived benefit of the education with either PRISM task or verbally (Control).

Comparing the values between the evaluation before and after the PRISM task (Test) or the flyer-based verbal briefing (Control), several significant differences were found (Table 5). Both groups showed a significant increase in the questions: “I see a relationship between EP and teeth” (PRISM: 1.60 ± 1.86 vs. 4.20 ± 1.24, *p* < 0.01; Control: 1.33 ± 1.83 vs. 4.43 ± 1.24, *p* < 0.01), “I see a relationship between EP and gums” (PRISM: 1.50 ± 1.88 vs. 4.05 ± 1.41, *p* < 0.01; Control: 1.05 ± 1.75 vs. 4.43 ± 1.15, *p* < 0.01) and “I see a relationship between EP and dental consultations” (PRISM: 2.18 ± 2.22 vs. 4.18 ± 1.57, *p* < 0.01; Control: 1.85 ± 2.09 vs. 4.23 ± 1.37, *p* < 0.01). Only in the PRISM group, the perception of the future EP as a foreign body reduced after the PRISM task (1.35 ± 1.83 vs. 0.58 ± 1.26, *p* = 0.01). Both groups perceived a similarly high benefit of the PRISM task (Test group) or the verbal briefing (Control group) and felt well-educated after the intervention (Figure 5). Thereby, the average benefit was experienced around 4.5 points on a scale between 0 and 5, whereby 5 was set as the maximal possible benefit.

## 4. Discussion

This current study revealed several results of practical interest. In part (I), patients prior to EP were found to have a high periodontal treatment need. Moreover, PRISM findings were not associated with oral health findings, but a gender effect was detected. The majority of patients perceived the importance of oral health and their EP as equal. Thus, the hypothesis that PRISM findings would be associated with oral health conditions was not confirmed. This implies, that the objective need for oral health treatment as assessed by a dentist and the subjective perceived need of patients is not correlated at all if patients do not get specific medical information about the importance of their dental health.

In part (II), i.e., the observational part of the current study, the hypothesis that both PRISM and verbal briefing would lead to a remarkable increase of awareness for oral health in patients before EP implantation could be confirmed. Thereby, the positive effect of both approaches was high and quite similar. In the PRISM group, a positive effect on the perception of the EP as a foreign body was noticed.

For the first time in literature, the visual tool PRISM has been introduced as a potential tool for oral health education. Only one previous study in the field of dental research used PRISM and assessed pain and suffering in patients with orofacial pain [21]. Therefore, the interpretation of the results with regard to available literature is limited. The first issue, which requires discussion is the patient cohort. In both cohorts of the study, the periodontal treatment need was high, with a range between 75 and 86% across sub-cohorts, and 30–43% had a potential dental focus. A previous study found similar results in patients before joint replacement, indicating that this cohort requires special dental attention [17]. Different explanations for the insufficient oral conditions of patients in the current study are conceivable. First, as shown in the Fifth German Oral Health Study (DMSV), periodontal treatment need of German general population is high [22]. Thus, high periodontal treatment need is an expectable result.

Second, patients before EP often suffer from chronic diseases and pain. This potentially affects their oral health behavior according to more complaint-oriented dental visit as a result of reduced awareness on oral health concerns, as observed for other groups of chronically ill patients [23]. Moreover, underlying diseases causing necessity of EP implantation could be of relevance; e.g., rheumatic diseases are related to oral and periodontal inflammation [24], potentially influencing the oral findings of the current study cohort, too. Altogether, increased attention in dental care appears reasonable in patients prior to EP; however, based on the low prevalence of EP infections (0.3–2% of patients, 6–13% of oral origin) [14,18], but, in contrast, the high prevalence of oral diseases in those patients might indicate an overestimation of the role of oral conditions in inducing EP infections. On the other hand, a variety of reports found a potential oral origin of the EP infections, which underlines the potential relevance of eliminating oral inflammation prior to EP implantation [15,25,26,27]. Therefore, the currently applied education approach to inform and sensitize patients prior to EP implantation for oral health issues can be summarized as reasonable and clinically relevant for this patient group.

To discuss now the potential of PRISM in this context, it is necessary to gain insight into this methodological concept, which is still largely unknown in the field of dentistry. Generally, PRISM is a visual tool, which is easy to understand for the patients [9] and has a good test-retest reliability [9,28] as well as satisfactory inter-rater reliability [9]. In principle, PRISM acts as a visual metaphor, following the principle of the so-called distance similarity metaphor [29], meaning that strengths of relationships between elements is visualized by their proximity on the board. The most important effect of using the metaphor is that it induces a reflection process, because patients are deliberating to explain their placement of the respective disks [7]. Thereby, the Relevance Theory of Metaphor can be mentioned [30], indicating that metaphors lead to a testing of patients’ own hypotheses and to interpret their own view in the role of an observer. It must thereby be considered that a metaphor can only be interpreted in a personally silent way and is therefore highly individual and is a special experience for the patient when solving the PRISM task [31]. Taken together, PRISM is a highly individual and active process, which is different from a “passive” educational verbal briefing, which is based on a flyer or checklist.

Based on this methodological background, the PRISM findings in the current study can be interpreted as: PRISM results differed between male and female only in one aspect in the cross-sectional study; male individuals rated the importance of EP more often higher than of oral health. This is not surprising, because health behavior is known to be affected by age and gender [32]. Interestingly, the independence of PRISM task and oral health indicates that the patient’s perception appears to be irrespective of their physical oral health situation. Considering the hypothesis of part (I), it might have been expectable that patients with worse oral health might also experience another relationship between their oral conditions and their selves on the PRISM board (e.g., higher distance between “My teeth” and “Myself”). As shown in the results, the distance between objects and subjects was quite small in the cross-sectional study part and majority of patients rated same importance for EP and oral health. This would indicate a strong relationship between oral health and patients, which is contradictory to the oral health findings. This ambivalence, however, could be one explanation for the positive effect of PRISM, as discussed in the following.

In part (II), the observational part of the current study, two forms of patient education were tested. For decades there exists a controversial discussion about, the appropriate and most successful way of patient education [33]. Especially for patients with systemic diseases, limited studies are available and successful oral health promotion activities are still questionable and needed [6,34]. Therefore, this current study compared PRISM as an activating method with a verbal briefing of an information leaflet as a rather a passive process. Both interventions showed positive effects on the perception of the importance of oral health and patients felt well-educated with both methods.

It is conceivable, that a major part of the effect in both methods is caused by the mere attention that is given to the patient and his or her oral health. Thus, the assessment of the benefit of the method itself is difficult to evaluate. However, and regardless of the interpretation of the results, it is remarkable that PRISM led to such a high perceived benefit for the patients although patients only placed the objects on the PRISM board, without receiving any specific information or distinct briefing on the importance of oral health for their EP. This indicates that the PRISM method itself induces a powerful self-reflection process [7].

Surprisingly, the majority of patients appear to place in the PRISM task their oral health issues and EP very close and in same distance. Even in this common pattern the visual metaphor appears to lead to an activation of patients in thinking about the relevance of their oral health. Although this is just an interpretation of the current study’s findings, this opens a new opportunity for active patient education and practitioner–patient communication. This supports one of the reported main strengths of PRISM methodology, inducing self-reflection and visualizing personally salient information to increase practitioner–patient-relationship building, [7]. When compared to the passive leaflet method, PRISM the reduced perception of EP as a foreign body, which indicates a further positive effect, which reaches beyond dentistry or oral health issues.

This is the first study that applied PRISM as a tool to support patient education in dental research. This novel and innovative methodological approach, which might offer a great opportunity and broad field of potential usage, is one major strength of the current study. The inclusion of more than 200 patients (122 patients (part I) and two groups with 40 patients each (part II),) which were prior to EP implantation is a further strength of the current study. Especially the balanced age, gender, and oral health conditions between all groups support the impact of the presented results. With the powerful and time-saving communication of relevant health information we could prove that the mere use of PRISM is as powerful as the passive communication is by means of a prepared information leaflet. This finding is of major clinical relevance and addresses a field of high interest. Finally, several limitations should be considered: The current study was only a short-term evaluation; long-term results and consequences on the patient’s oral health and dental behavior will be needed to reveal the measurable benefit of the use of PRISM. For evaluation only questionnaires were used, which is also a limiting factor. PRISM requires a well-educated interviewer and a very well-structured usage; this limits the generalizability of the findings. To reveal transferable results, multicentric approaches, introducing multiple interviewers would be recommendable in the future. The current study is rather a proof of concept and must therefore be interpreted with caution. This must also be seen in context of sample size in part (II). While the current study was based on a sample size estimation, an appropriate sample size calculation based on the current study’s findings should be performed in future investigations in the field. Therefore, the current findings should be seen as preliminary results. Reliable data will be needed to further support the findings and to clarify, what potential application PRISM will have in the field of dental outcome research, patient information, and clinician–patient communication. The current study provided several interesting results in a highly relevant cohort, showing high oral disease burden in patients prior to EP. Regardless, oral health and related education are important in all segments of life, making the approach also potentially interesting for all groups of dental patients. Therefore, future studies should consider the PRISM approach in other patient groups.

## 5. Conclusions

Patients prior to EP show high periodontal treatment need, potential dental foci, and need for education. PRISM is a novel visual tool that can support patient education by inducing a self-reflection process. The use of PRISM has comparable information effects as a flyer-based verbal briefing and shows further benefits beyond oral health issues. Further studies in dental research are required to assess long-term effects of PRISM on clinical outcomes.

## Figures and Tables

**Table 1:** Patient characteristics cross-sectional part of the study (*n* = 122).

	Patients before Implantation of EP (*n* = 122)
Gender (female in % (*n*))	46.7% (57)
Age in years (mv ± sd)	66.38 ± 11,42
Number of remaining teeth (mv ± sd)	18.66 ± 8.42
Dental treatment need (% (*n*))	24.6% (30)
Periodontal treatment need (% (*n*))	86.1% (105)
Presence of potential dental focus (% (*n*))	43.4% (53)

**Table 2:** Findings of the PRISM task across participants.

		Patients before Implantation of EP (*n* = 122)
Importance of healthy teeth (in mm, mv ± sd (median, range))		11.0 ± 33.9 (0, 0–170)
Importance of healthy gums (in mm, mv ± sd (median, range))		11.8 ± 34.4 (0, 0–169)
Importance of consulting dentist (in mm, mv ± sd (median, range))		13.4 ± 42.0 (0, 0–209)
Cluster relationship of EP and teeth (% (*n*))	Same importance of EP and teeth	76.2% (93)
	Teeth more important than EP	11.5% (14)
	EP more important than teeth	12.3% (15)

**Table 3:** Cross-sectional study (*n* = 122): *p*-values for the associations between PRISM results and age, gender, as well as oral health conditions.

	Importance of Healthy Teeth	Importance of Healthy Gums	Importance of Consulting the Dentist	Relationship EP and Teeth
Age	0.76 ^a^	0.68 ^a^	0.64 ^a^	0.16 ^b^
Gender	0.68 ^c^	0.57 ^c^	0.09 ^c^	0.02 ^b^
Number of teeth	0.04 ^a^	0.41 ^a^	0.29 ^a^	0.26 ^b^
Dental treatment need	0.46 ^c^	0.19 ^c^	0.56 ^c^	0.56 ^b^
Periodontal treatment need	0.64 ^c^	0.45 ^c^	0.12 ^c^	0.76 ^b^
Potential dental focus	0.62 ^a^	0.74 ^a^	0.61 ^a^	0.86 ^b^

^a^ Kruskal-Wallis test, ^b^ chi-square test, ^c^ Mann-Whitney-U test, significance level *p* < 0.05.

**Table 4:** Characteristics of participants who received the education either by PRISM (PRISM group) or verbally using a flyer (Control group) during observational study.

	PRISM (*n* = 40)	Control (*n* = 40)	*p*-Value
Gender (female in % (*n*))	55.0% (22)	47.5% (19)	0.67
Age in years (mv ± sd)	69.78 ± 11.02	70.02 ± 11.04	0.87
Number of remaining teeth (mv ± sd)	21.10 ± 8.09	19.10 ± 8.63	0.29
Dental treatment need (% (*n*))	42.5% (17)	45.0% (18)	0.99
Periodontal treatment need (% (*n*))	80.0% (32)	75.0% (30)	0.79
Presence of a potential dental focus (% (*n*))	30.0% (12)	30.0% (12)	0.99

**Table 5:** Differences in different questions before and after education with either PRISM task or verbal education combined with flyer during observational study part. Values are on a scale between 0 (very low/not at all/does not comply) and 5 (very high/very much/does fully comply).

Question	PRISM (*n* = 40)			Control (*n* = 40)		
	Before	After	*p*-Value	Before	After	*p*-Value
I perceive EP as a foreign body	1.35 ± 1.83	0.58 ± 1.26	0.01	1.35 ± 1.76	1.10 ± 1.69	0.36
Oral health has high relevance in my life	4.28 ± 1.41	4.55 ± 1.15	0.12	4.50 ± 1.15	4.70 ± 0.65	0.34
My oral health status is a problem	1.20 ± 1.62	0.88 ± 1.40	0.23	1.42 ± 1.95	1.35 ± 1.94	0.76
My teeth are a disruptive factor	0.59 ± 1.41	0.34 ± 0.94	0.23	0.79 ± 1.82	0.72 ± 1.62	0.81
I see a relationship between EP and teeth	1.60 ± 1.86	4.20 ± 1.24	<0.01	1.33 ± 1.83	4.43 ± 1.24	<0.01
I see a relationship between EP and gums	1.50 ± 1.88	4.05 ± 1.41	<0.01	1.05 ± 1.75	4.43 ± 1.15	<0.01
I see a relationship between EP and dental consultations	2.18 ± 2.22	4.18 ± 1.57	<0.01	1.85 ± 2.09	4.23 ± 1.37	<0.01
Since I need an EP, I performed oral hygiene less intensively	0.65 ± 1.41	0.65 ± 1.37	0.99	0.40 ± 1.19	0.73 ± 1.54	0.19
Since I need an EP, I visited the dentist less often for prevention	0.68 ± 1.56	0.77 ± 1.51	0.72	1.05 ± 1.96	1.20 ± 1.95	0.82
Since I need an EP, I have less energy to perform oral hygiene	0.37 ± 1.13	0.70 ± 1.44	0.12	0.63 ± 1.35	1.05 ± 1.80	0.13
Since I need an EP, oral health has a lower importance in my life	0.60 ± 1.39	0.85 ± 1.67	0.29	0.63 ± 1.53	0.93 ± 1.83	0.42

## Data Availability

The datasets used and/or analysed during the current study available from the corresponding author on reasonable request.
